# Effect of the valence state of Mn in MnO_*x*_/Ti_4_O_7_ composites on the catalytic performance for oxygen reduction reaction and oxygen evolution reaction†

**DOI:** 10.1039/d0ra08575h

**Published:** 2021-01-05

**Authors:** Fan Bai, Lincheng Xu, Daode Wang, Li An, Zhanzhong Hao, Fan Li

**Affiliations:** Beijing Key Laboratory for Catalysis and Separation, Department of Environment and Chemical Engineering, Beijing University of Technology Beijing 100124 China vanadiumli@bjut.edu.cn; Department of Chemistry, Baotou Teachers' College Baotou 014000 China 18438602389@163.com; Beijing Key Laboratory for Catalysis and Separation, Faculty of Environment and Life, Beijing University of Technology Beijing 100124 China 08131@bjut.edu.cn

## Abstract

Manganese oxide composites with mixed valence states were prepared through the hydrothermal method by compositing with Ti_4_O_7_ and calcining at different temperatures, and their ORR and OER catalytic performance were investigated. The prepared catalysts were characterized by XRD, SEM-EDS, HRTEM-EDS, and XPS methods to analyse their phase constitution, morphology feature, and surface composition. The major phase of manganese oxides was Mn_3_O_4_, which is a one-dimensional structure, and its growth was induced by Ti_4_O_7_. The ORR and OER catalytic activity can be enhanced due to the preferred orientation of manganese oxides. Electrochemical measurements, namely CV, LSV and EIS, were utilized for determining the ORR and OER catalytic activity, whereas CA and ADT were used for studying the durability and stability. A Li–O_2_ battery was assembled to test the electrochemical behavior and properties in practical application. MnO_*x*_/Ti_4_O_7_ calcined at 300 °C exhibited the best catalytic activity of 0.72 V *vs.* RHE half-wave potential for ORR and 0.67 V *vs.* RHE overpotential for OER. The proportion of Mn^3+^ was also highest in all the MnO_*x*_/Ti_4_O_7_ composites. The assembled Li–O_2_ battery shows high performance with a voltage gap of only 0.85 V. Therefore, it can be affirmed that the inducement of Ti_4_O_7_ could strengthen the preferred orientation in manganese oxide growth and Mn^3+^ in MnO_*x*_/Ti_4_O_7_ plays a vital role in catalyzing ORR and OER, with both improving the ORR and OER bifunctional catalytic performance of manganese oxides.

## Abstract 2

Manganese oxide composites with mixed valence states were prepared by compositing with Ti_4_O_7_ and calcination temperature could influence their ORR and OER catalytic performance observably.

## Graphical abstract

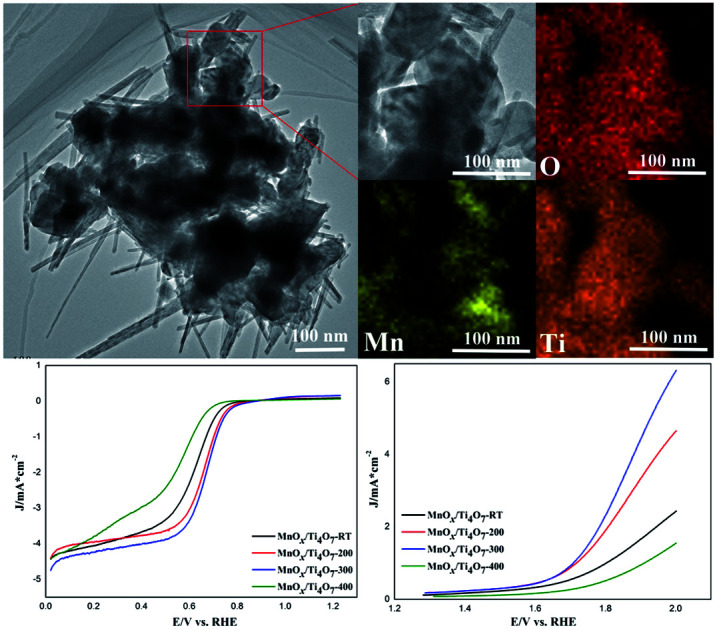

## 1. Introduction

With the rapid development of the economy and society, the increase in energy demand leads to a shortage of resources, as well as an increase in environmental pollution and other serious problems. Therefore, it is very important to seek a clean, safe, and efficient energy system for solving this problem.^1^ With the advancement of research, fuel cells, metal–air batteries, and other new energy batteries have attracted much attention due to their excellent performance.^2–4^ However, the sluggish kinetics of the oxygen reduction reaction (ORR) and oxygen evolution reaction (OER) at the oxygen electrode restricts their development severely; so it has become the most urgent problem to develop an efficient ORR and OER bifunctional catalyst.^5,6^

Traditionally, noble metals, such as Pt and Ir, have performed high ORR and OER bifunctional catalytic activity and have been widely used.^7–11^ However, their large-scale application is limited due to their scarcity and high cost. Therefore, transition metal oxides have become one of the best alternatives due to their low price, abundant reserves, and high-efficiency catalytic performance.^12–16^ Manganese is one of the transition metals, and the abundance of the element is only next to iron and titanium. The valence electron configuration is 3d^5^4s^2^, which leads to various valence states and hence could form a series of oxides.^17^ As for manganese oxides, different species could exist steadily in the pH range of −2 to 16 at a certain suitable potential. A flexible valence and crystal structure further result in different physicochemical properties.^18^ Therefore, it is possible to utilize manganese oxides in electrocatalysis.

Numerous researches have been carried out regarding the synthesis and electrocatalytic property analysis of various manganese oxides, such as MnO_2_, Mn_2_O_3_, Mn_3_O_4_, and so on.^19–22^ Most of them have been proved to be efficient ORR/OER catalysts. The influence of crystal structure and valence state on electrochemical performance is becoming clear with intensive study. [MnO_6_] octahedron, which is the basic unit of manganese oxides, could connect each other through different ways and produce a variety of species. Therefore, MnO_2_ can form a series of crystals as well as exhibit different ORR/OER catalytic properties, capacitance characteristics, and energy storage properties.^23–26^ Certain specific lattice planes, such as (211) for α-MnO_2_, are also precisely controlled, and hence efficient catalytic property could be achieved by optimizing preparation technology.^27,28^ It has been proved that Mn^3+^ possesses optimal ORR and OER catalytic activity, which is explained theoretically by Suntivich *et al.*^29,30^ However, Mn_3_O_4_ of pure phase, in which Mn^3+^ occupies the [MnO_6_] octahedron site, shows poor OER catalytic activity due to Jahn–Teller distortion,^31^ which implies that it is the interaction of the crystal structure and valence state that controls the catalytic performance of manganese oxides.

In this work, manganese oxides were synthesized through the hydrothermal method and calcined at different temperatures to investigate the influence of the valence state. Besides, the role of Ti_4_O_7_ on adjusting the crystal structure and preferred orientation was also studied. The interaction of relevant factors was analysed synthetically to investigate the ORR and OER bifunctional catalytic performance of manganese oxides and their potential application prospects.

## 2. Experimental section

### 2.1 Synthesis of sample

Manganese oxides were prepared by the hydrothermal method and roasted further. 1.25 mmol KMnO_4_ (>99%, Tongguang Fine Chemicals) and 25 mmol carbon powder XC-72 (>99%, Alfa Aesar) were dissolved in 40 mL deionized water under magnetic stirring for 10 min. Three drops of ammonium hydroxide (25–28%, Fuchen Chemicals) were added to adjust pH ≈ 9. Then, 4 mL ethylene glycol (>95%, Tongguang Fine Chemicals) was added immediately, and magnetically stirred for 20 min. At this time, Ti_4_O_7_ (>95%, Titanium Energy Technology) could be added (a) 1.25 mmol and magnetically stirred for another 20 min or (b) none as the control group. Both (a) and (b) were then transferred into a Teflon-lined stainless steel autoclave and kept at 120 °C for 24 h. Finally, the prepared sample was collected by centrifugation, washed with deionized water and anhydrous ethanol successively and dried in a vacuum oven at 80 °C for 12 h. The obtained samples (a) and (b) were named MnO_*x*_/Ti_4_O_7_ and MnO_*x*_, respectively. Sample (a) was further subjected to thermal treatment in synthetic air at different calcination temperatures ranging from room temperature to 400 °C and named as MnO_*x*_/Ti_4_O_7_-RT, MnO_*x*_/Ti_4_O_7_-200, MnO_*x*_/Ti_4_O_7_-300, and MnO_*x*_/Ti_4_O_7_-400, respectively.

### 2.2 Characterization methods

The phase analysis of the powder samples was performed with X-ray diffraction (XRD, Bruker D8 advance) at a scan rate of 10 deg min^−1^ from 10° to 80°. The morphology and microstructure were studied by scanning electron microscopy (SEM, JEOL JSM-7900) and high-resolution transmission electron microscopy (HRTEM, FEI Tecnica G2 F20). The valence state analysis of the surface was carried out by X-ray photoelectron spectroscopy (XPS, ESCALAB 250Xi) with Al Kα radiation source.

### 2.3 Electrochemical measurements

A three-electrode system, including a glassy carbon working electrode of diameter *Φ* = 5 mm controlled by the rotating disk electrode (RDE, Pine AFMSRCE), a Hg/HgO in 0.1 mol L^−1^ KOH reference electrode, and a carbon rod counter electrode, was used in cyclic voltammetry (CV), linear sweep voltammetry (LSV), and electrochemical impedance spectroscopy (EIS) tests. An electrochemical workstation (Metrohm Auto-Lab PGSTAT302N) was used for setting and regulating the parameters. The electrode material was prepared by dispersing 2.0 mg powder sample in 1 mL anhydrous ethanol and 0.5 mL 0.2% Nafion ethanol solution. Subsequently, the mixture was converted into a slurry through an ultrasonic dispersing instrument. 20 μL of the electrode material slurry, which could divided into 4–6 times equally, was added dropwise onto the glassy carbon working electrode with a microsyringe. The three-electrode system was installed in a customized electrolytic cell and submerged in 0.1 mol L^−1^ KOH solution saturated with argon for activation and then saturated in oxygen for electrochemical measurements. As for the accelerated durability test (ADT), chronoamperometry (CA) and polarization curve tests, a glassy carbon piece of 10 × 10 mm was used as the working electrode. The formulation of the electrode material slurry was invariant but the amount was changed to 200 μL.

### 2.4 Li–O_2_ battery tests

The powder sample, conductive carbon black (Super P, >99%, Alfa Aesar) and agglomerant (polyvinylidene fluoride, PVDF) were mixed at a mass ratio of 3 : 6 : 1. *N*-Methyl-2-pyrrolidinone (NMP, >99%, Aladdin) was then added to configure the slurry. The carbon paper was used as a current collector substrate for spraying the slurry. The whole electrode material was dried at 120 °C for 12 h in an oven and then cut into a circular piece of 14 mm diameter for assembling the battery. Finally, the type CR2032 coin lithium–oxygen battery was assembled in an argon-filled glove box (Braun, Lab Star) and tested in a customized pure oxygen-filled battery box for the charge and discharge test (Neware, CT-4008).

## 3. Results and discussion

### 3.1 Phase identification and morphology analysis

**Fig. 1:**
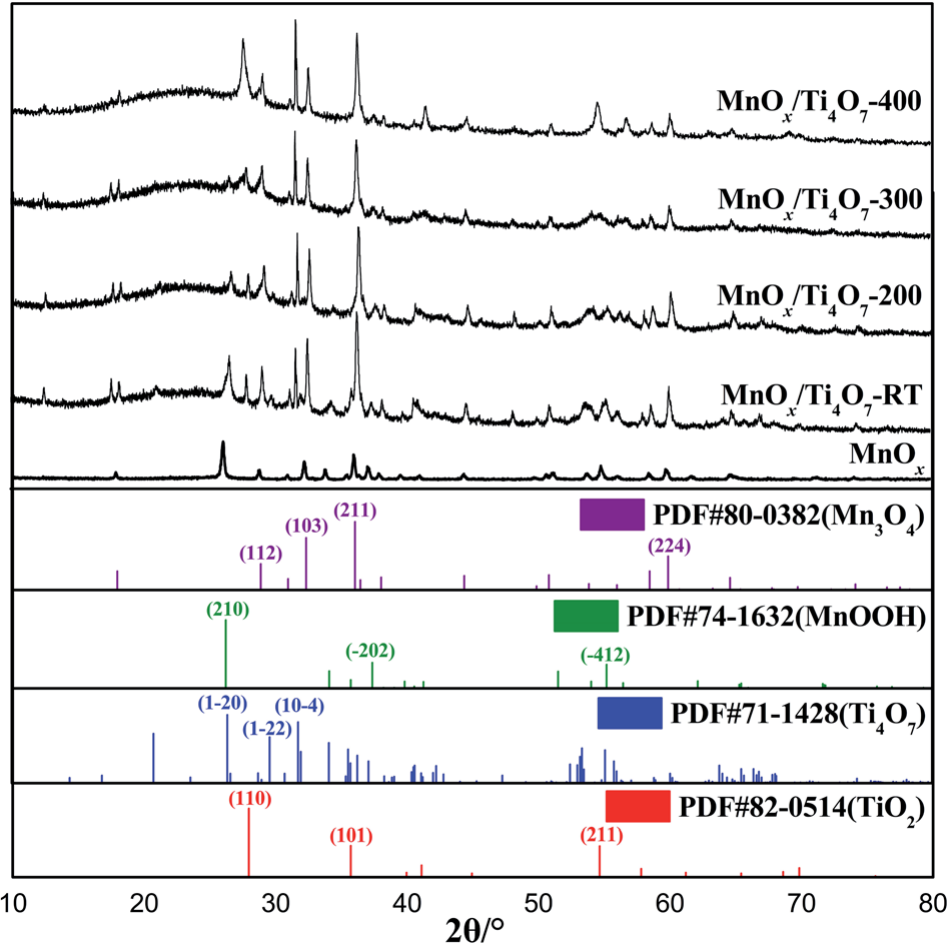
XRD patterns of MnO_*x*_ and MnO_*x*_/Ti_4_O_7_ at different calcination temperatures.

**Fig. 2:**
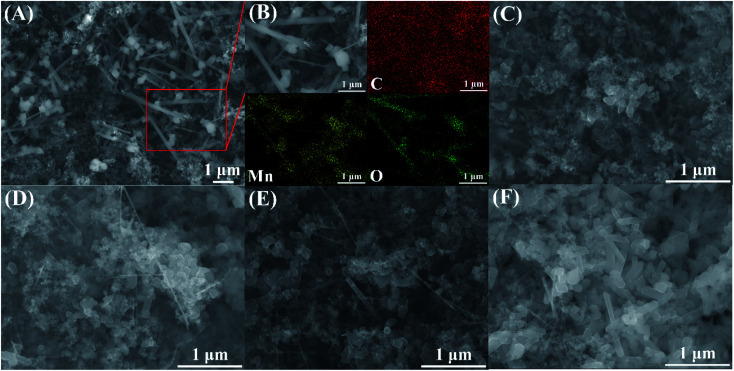
(A) SEM and (B) EDS images of MnO_*x*_, and SEM images of (C) MnO_*x*_/Ti_4_O_7_-RT, (D) MnO_*x*_/Ti_4_O_7_-200, (E) MnO_*x*_/Ti_4_O_7_-300, and (F) MnO_*x*_/Ti_4_O_7_-400.

Fig. 1 shows the XRD patterns of MnO_*x*_ powder samples without any further treatment and MnO_*x*_/Ti_4_O_7_ powder samples obtained at different calcination temperatures in air. The peaks at 2*θ* = 28.9°, 32.4°, 36.1°, and 59.9° were assigned to (112), (103), (211), and (224) crystal planes of Mn_3_O_4_ (PDF #80-0382), and peaks at 2*θ* = 26.3°, 37.4°, and 55.2° were ascribed to (210), (−202), and (−412) crystal planes of MnOOH (PDF #74-1632). Similarly, for MnO_*x*_/Ti_4_O_7_ powder samples, the XRD patterns can be assigned to Mn_3_O_4_ (PDF #80-0382), MnOOH (PDF #74-1632), Ti_4_O_7_ (PDF #71-1428), and rutile TiO_2_ (PDF #82-0514). It is worth noting that the phase of both manganese oxides and titanium oxides do not maintain uniformity following the change of calcination temperature. The sample without further thermal treatment shows an obvious MnOOH (210) crystal plane diffraction peak, and this peak weakens gradually by the rise of calcination temperature until it disappears completely at 400 °C.^32^ As for titanium oxides, the corresponding peak of rutile TiO_2_ shows noteworthy enhancement at 400 °C.^33,34^ Therefore, it can be affirmed that Ti_4_O_7_ was oxidized to TiO_2_, which may lead to a different microstructure morphology at 400 °C, as shown in Fig. 2. In addition, it is noticed that the XRD peaks of Mn_3_O_4_ and Ti_4_O_7_ generate a certain degree of overlap, which results in the induction of manganese oxide growth by Ti_4_O_7_.

**Fig. 3:**
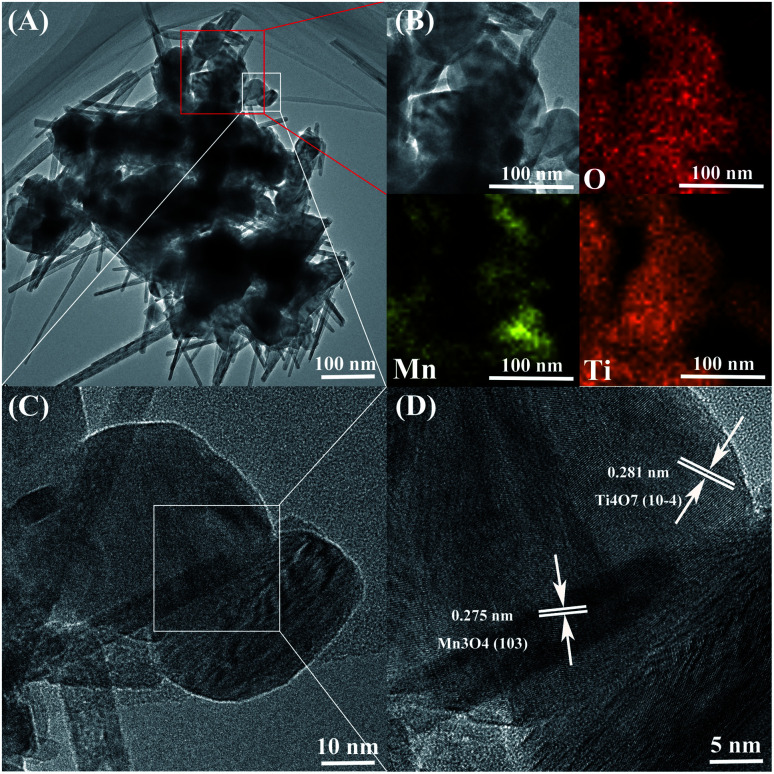
(A) TEM, (B) EDS, (C and D) HRTEM images of MnO_*x*_/Ti_4_O_7_.

**Fig. 4:**
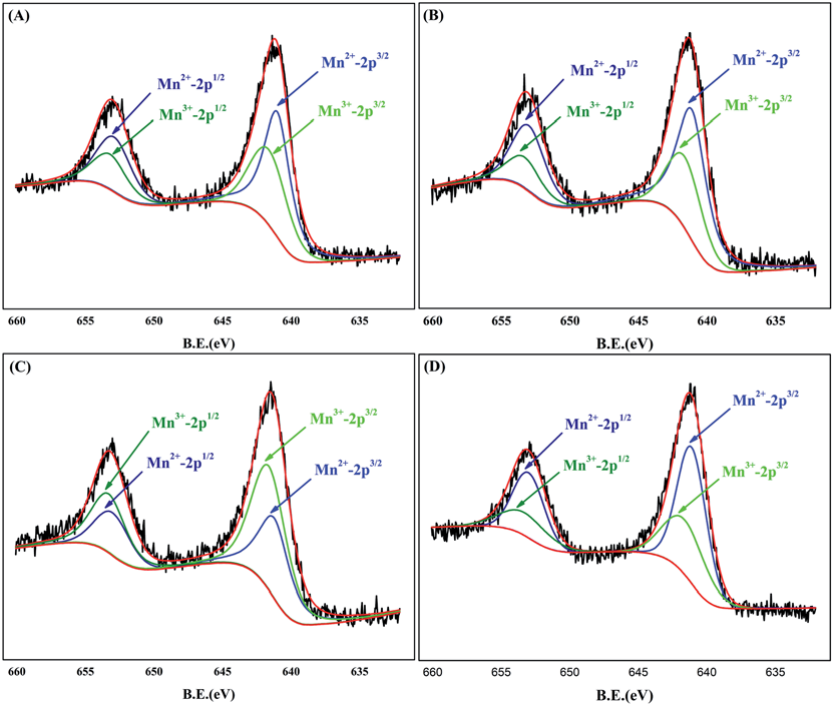
XPS patterns for Mn 2p of samples (A) MnO_*x*_/Ti_4_O_7_-RT, (B) MnO_*x*_/Ti_4_O_7_-200, (C) MnO_*x*_/Ti_4_O_7_-300 and (D) MnO_*x*_/Ti_4_O_7_-400.

SEM and energy dispersive spectrum (EDS) mapping show the microstructure and elemental distribution of MnO_*x*_. According to Fig. 2(A) and (B), MnO_*x*_ appears as a one-dimensional structure with a diameter of 20–150 nm or nanoparticles of grain size about 60–200 nm, which differs from Ti_4_O_7_ particle in morphology feature and crystallite dimension as shown in Fig. S1.† However, the morphology feature of MnO_*x*_ changes into a slimmer one-dimensional nanostructure when composed with Ti_4_O_7_. According to Fig. S2(A) and (B),† the high-magnification SEM images of MnO_*x*_ and MnO_*x*_/Ti_4_O_7_ show obvious variation in the diameter of MnO_*x*_, which is due to the introduction of Ti_4_O_7_. The fine structure and interaction relationship between MnO_*x*_ and Ti_4_O_7_ can be further proved by TEM images in Fig. 3 and S3.† It can be found that MnO_*x*_ and Ti_4_O_7_ combine together and exhibit partial overlapping with another. The inducement of Ti_4_O_7_ influences the MnO_*x*_ morphology feature and may further strengthen the preferred orientation of manganese oxides. Besides, it can be observed in SEM images that the morphology of the samples also reveals similar results like XRD patterns. There are several common morphology features in MnO_*x*_/Ti_4_O_7_-RT, MnO_*x*_/Ti_4_O_7_-200, and MnO_*x*_/Ti_4_O_7_-300 samples, as shown in Fig. 2(C–E). Manganese oxide exhibits a one-dimensional structure of about 30 nm diameter and a disordered arrangement in all directions, whereas Ti_4_O_7_ exhibits a particle morphology. The introduction of Ti_4_O_7_ strengthens the preferred orientation in manganese oxide growth and makes the diameter distribution more homogeneous.^35,36^ After calcination at 400 °C for 2 h, spindle-shaped manganese oxide turns into a wider shape of about 100 nm according to Fig. 2(F). It is considered that the Ostwald ripening mechanism plays an important role in the growth of manganese oxide crystals.^37^ Moreover, according to XRD patterns, the formation of the rutile TiO_2_ phase was also observed when calcined at 400 °C. Therefore, it is essential to perform the XPS analysis in order to characterize the chemical composition on the surface of the samples at different calcination temperatures (Fig. 4). The mixed valence states of manganese can be assigned to Mn^2+^ and Mn^3+^ according to the phase composition of Mn_3_O_4_ and MnOOH. The ratio of Mn^3+^/Mn^2+^ is diverse from each other and reaches a maximum value at a calcination temperature of 300 °C.

### 3.2 Electrochemical properties and analysis

The electrochemical properties of MnO_*x*_ and MnO_*x*_/Ti_4_O_7_ samples were tested in 0.1 mol L^−1^ KOH aqueous solution. For CV and LSV tests, the electrolyte solution was first saturated with argon in order to activate the catalytic material and then saturated with oxygen. For EIS, ADT, and CA tests, the electrolyte solution was saturated with oxygen and then aerated continuously. All the electrochemical measurements in this work were carried out without *iR* compensation.

**Fig. 5:**
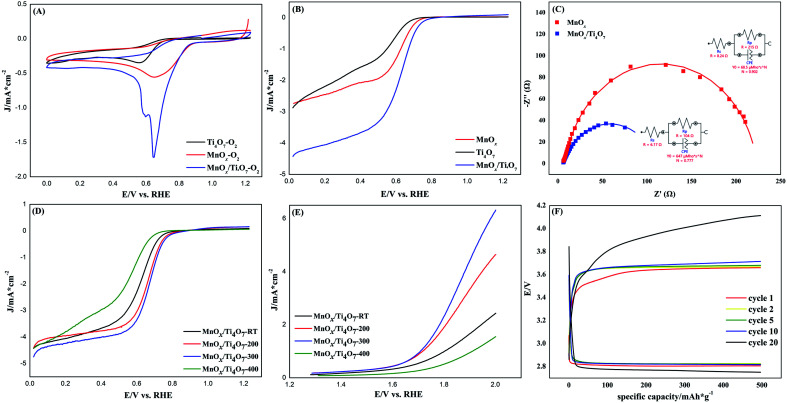
(A) CV and (B) LSV curves of MnO_*x*_, Ti_4_O_7_, and MnO_*x*_/Ti_4_O_7_, at a scan rate of 10 mV s^−1^; (C) EIS tests and equivalent circuit of MnO_*x*_ and MnO_*x*_/Ti_4_O_7_; LSV curves for (D) ORR and (E) OER of MnO_*x*_/Ti_4_O_7_-RT, MnO_*x*_/Ti_4_O_7_-200, MnO_*x*_/Ti_4_O_7_-300, and MnO_*x*_/Ti_4_O_7_-400, at a scan rate of 10 mV s^−1^; all above tests were carried out in O_2_-saturated 0.1 mol L^−1^ KOH electrolyte; (F) cycling performance of the Li–O_2_ battery assembled with MnO_*x*_/Ti_4_O_7_-300.

As shown in Fig. 5(A), the CV test was processed to evaluate the ORR performance. MnO_*x*_ shows a relatively stronger peak and lower overpotential than the sample with only Ti_4_O_7_. The reductive peak of MnO_*x*_/Ti_4_O_7_ is worthy of further studies for its specific double-peak pattern. By using the standard electrode potential of manganese element as a reference, the peak of 0.59 V *vs.* RHE can be assigned to the reduction reaction of Mn_3_O_4_ to Mn(OH)_2_, and 0.65 V *vs.* RHE can be ascribed to the ORR. Meanwhile, the ORR peak shows a high current density and onset potential. The LSV curves at 1600 rpm present the ORR performance by half-wave potential and limiting diffusion current density and OER performance by overpotential more directly according to Fig. 5(B) and S4.† It is obvious that the MnO_*x*_/Ti_4_O_7_ catalytic material exhibits far better ORR and OER catalytic activity than MnO_*x*_ or Ti_4_O_7_, which can be attributed to the composition of manganese oxides and Ti_4_O_7_, as shown above in the SEM image in Fig. 2 and S2.† The EIS test was performed to analyse the charge transfer properties of MnO_*x*_ and MnO_*x*_/Ti_4_O_7_, as shown in Fig. 5(C). The sample MnO_*x*_/Ti_4_O_7_ shows a smaller charge transfer resistance (104 Ω) than that of MnO_*x*_ (215 Ω), indicating a faster charge transfer kinetics process. Manganese oxide grows in a one-dimensional direction supported by Ti_4_O_7_ particle, which relieves the intrinsic low conductivity of manganese oxides enhancing the ORR performance of the MnO_*x*_/Ti_4_O_7_ composite material.

Moreover, the introduction of Ti_4_O_7_ can also promote the catalysis durability of the ORR according to CA curves in Fig. S5.† The current of MnO_*x*_ and MnO_*x*_/Ti_4_O_7_ reduces about 50.1% and 39.4%, respectively, after 10 000 s. It is concluded that the preferred orientation of MnO_*x*_ induced by Ti_4_O_7_ will not only enhance the ORR/OER catalytic activity but also be beneficial to the durability. This powerful inducement was derived from the lattice intergrowth of Mn_3_O_4_ and Ti_4_O_7_, according to XRD patterns at around 32°, which can also be proved by HRTEM and EDS in Fig. 3. The elements manganese and titanium exhibit fractal features, as shown in Fig. 3(A) and (B). The HRTEM images exhibit obvious lattice fringes, which can be assigned to the (103) crystal plane of Mn_3_O_4_ and (10−4) crystal plane of Ti_4_O_7_, according to Fig. 3(D) and S3.† These two XRD pattern peaks overlap with each other to a certain degree. It can be affirmed that the growth of Mn_3_O_4_ was induced by Ti_4_O_7_ and produced preferred orientations. Besides, this inducement of Ti_4_O_7_ could also enhance the catalytic activity of the sample MnO_*x*_/Ti_4_O_7_ by increasing the number of active sites. ECSA tests were performed to characterize the same and the results are shown in Fig. S6 and S7.† The current data (*i*) was obtained from CV tests at a scan rate of 10, 20, 40, 60, 80, and 100 mV s^−1^, and the slope of the *i*–*ν* pattern could be used to describe ECSA. The corresponding ECSA value was calculated as 48.6 cm^2^ and 39.7 cm^2^ for MnO_*x*_/Ti_4_O_7_ and MnO_*x*_, respectively, indicating an increase of about 22.4% due to the inducement of Ti_4_O_7_.

On the basis of the above research, the influence of the calcination temperature on electrocatalysis was studied. The ORR and OER bifunctional catalytic performance of MnO_*x*_/Ti_4_O_7_-RT, MnO_*x*_/Ti_4_O_7_-200, MnO_*x*_/Ti_4_O_7_-300, and MnO_*x*_/Ti_4_O_7_-400 are shown in Fig. 5(D) and (E). The results exhibit completely consistent rules for both ORR and OER. The half-wave potential and limiting diffusion current density for ORR and the overpotential for OER all move in the direction of better catalytic performance along with the rise of calcination temperature until 300 °C. The comparison of electrochemical properties of the materials synthesized in this work and that of other studies are shown in Table S1.† However, when the calcination temperature reaches 400 °C, the catalytic property for both ORR and OER reveals a distinct decline, even worse than that of MnO_*x*_/Ti_4_O_7_-RT. The corresponding K–L equation and Tafel curves could also prove the decline in the catalytic performance from electron transfer numbers and kinetics, as shown in Fig. S8 and S9.† The MnO_*x*_/Ti_4_O_7_-400 sample exhibits electron transfer numbers of 3.68 for ORR and Tafel slope of −147 and 413 mV dec^−1^ for ORR and OER, respectively, which indicates poorer electron transfer numbers and reaction kinetics than MnO_*x*_/Ti_4_O_7_ samples at other calcination temperature. The XRD patterns showed an evident diffraction peak of TiO_2_ (110) crystal plane at 400 °C, which could result in the decline of conductivity and catalytic activity.^38,39^ By comparing the SEM images shown in Fig. 2(C–E) with Fig. 2(F), it can be found that the morphology of manganese oxide changes evidently. The one-dimensional structure becomes wider in diameter and shorter in length. The decrease in the number of crystal planes with high catalytic activity may be the main reason for low electron transfer numbers and high Tafel slope, resulting in ORR and OER catalytic performance attenuation.

In addition, the catalytic stability of MnO_*x*_/Ti_4_O_7_ catalysts at different calcination temperatures was investigated by ADT, as shown in Fig. S10.† After 5000 cycles of CV test, all the samples exhibited a decline to a certain extent, especially MnO_*x*_/Ti_4_O_7_-400. The corresponding polarization curves in Fig. S10† indicates that MnO_*x*_/Ti_4_O_7_-400 exhibits poorer ORR and OER catalytic activity than any other samples, both before and after ADT. On the contrary, MnO_*x*_/Ti_4_O_7_-300 shows the best performance.

XPS analysis was used to indicate the valence state of the sample surface. The narrow sweep of manganese element was limited to the binding energy of 632–660 eV, as shown in Fig. 4(A–D). The Mn 2p spectrum includes two main regions, and each can be divided into two peaks of Mn 2p_1/2_ and Mn 2p_3/2_, respectively.^40^ According to the NIST database of XPS and the previous results of XRD patterns, valence states of MnO_*x*_/Ti_4_O_7_ samples were assigned to Mn^3+^ (2p_3/2_ for 641.6 eV and 2p_1/2_ for 653.3 eV) and Mn^2+^ (2p_3/2_ for 641.1 eV and 2p_1/2_ for 653.0 eV). The valence state of samples changes to a certain degree, according to Fig. 4, as a result of the phase composition transformation in XRD patterns. The peak area and ratio of Mn^3+^/Mn^2+^ are listed in Table 1 for comparing samples at different calcination temperatures. The proportion of Mn^3+^ increases continuously from room temperature to 300 °C but declines at 400 °C, which is consistent with the ORR and OER catalytic performance completely. Therefore, the location of Mn^3+^ was considered as the catalytically active site for ORR and OER. This can be explained by the research of Suntivich *et al.*^29,30^ According to their study, the catalytic performance for ORR and OER depends on two key factors, which are the amount of electron occupied in the σ*-antibonding orbital (e_g_) and the covalent property of M–O (transition metal–oxygen). When the value of e_g_-filling is ∼1, the covalency between the transition metal 3d and oxygen 2p orbitals can be further increased, which leads to maximum ORR and OER catalytic activity ultimately. As for Mn^3+^ existing in oxygen-octahedron, the outermost electron configuration was d^4^ with a high spin configuration, which resulted in the value of e_g_-filling equivalent to 1. Thus, high-efficiency bifunctional catalytic performance of manganese can be interpreted by this theory.

**Table 1:** The valence state analysis of MnO_*x*_/Ti_4_O_7_ samples at different calcination temperatures obtained from XPS

Sample name	Orbit	Binding energy/eV	Area	Ratio of Mn^3+^/Mn^2+^
MnO_*x*_/Ti_4_O_7_-RT	Mn^3+^-2p_3/2_	641.6	10 002	**0.699**
	Mn^2+^-2p_3/2_	641.1	14 306	
	Mn^3+^-2p_1/2_	653.3	5001	
	Mn^2+^-2p_1/2_	653.0	7153	
MnO_*x*_/Ti_4_O_7_-200	Mn^3+^-2p_3/2_	641.6	6842	**0.530**
	Mn^2+^-2p_3/2_	641.1	12 901	
	Mn^3+^-2p_1/2_	653.3	3421	
	Mn^2+^-2p_1/2_	653.0	6451	
MnO_*x*_/Ti_4_O_7_-300	Mn^3+^-2p_3/2_	641.6	12 850	**1.552**
	Mn^2+^-2p_3/2_	641.1	8280	
	Mn^3+^-2p_1/2_	653.3	6425	
	Mn^2+^-2p_1/2_	653.0	4140	
MnO_*x*_/Ti_4_O_7_-400	Mn^3+^-2p_3/2_	641.6	6939	**0.567**
	Mn^2+^-2p_3/2_	641.1	12 231	
	Mn^3+^-2p_1/2_	653.3	3469	
	Mn^2+^-2p_1/2_	653.0	6115	

### 3.3 Li–O_2_ battery performance

The top-performance bifunctional catalytic material MnO_*x*_/Ti_4_O_7_-300 was chosen for assembling the battery. The Li–O_2_ battery performance test was performed at a constant current density of 100 mA g^−1^ and a constant capacity of 500 mA h g^−1^. As shown in Fig. 5(F), the charge/discharge voltage plateau of the first cycle was about 3.65 V and 2.80 V, which remains almost stable for 10 cycles. The voltage gap was only 0.85 V, which is attributed to the excellent ORR and OER bifunctional catalytic performance of the catalytic material. However, the voltage gap increases from cycle 10. After 20 cycles, the charge/discharge voltage plateau reaches 4.10 V and 2.76 V, which represents the attenuation of catalytic performance. The catalytic stability in battery cycle is to be further improved.

## 4. Conclusion

Transition metal manganese oxides have attracted extensive attention from researchers benefiting from the low cost, abundant reserve, easy synthesis, and high-efficiency catalytic performance, especially in the electrocatalysis domain. A series of manganese oxides were synthesized at different calcination temperatures, and several conclusions can be drawn from this study.

(1) The composition of Ti_4_O_7_ was beneficial to improving the catalytic performance of manganese oxides. By comparing MnO_*x*_ and MnO_*x*_/Ti_4_O_7_ samples, it was observed that the presence of Ti_4_O_7_ not only improves the intrinsic low conductivity of manganese oxides but also induces manganese oxide growth in a one-dimensional direction to form a preferred orientation, which enhances the ORR and OER bifunctional catalytic performance together.

(2) The ratio of Mn^3+^ impacts the catalytic performance significantly. By controlling the calcination temperature, MnO_*x*_/Ti_4_O_7_-RT, MnO_*x*_/Ti_4_O_7_-200, MnO_*x*_/Ti_4_O_7_-300, and MnO_*x*_/Ti_4_O_7_-400 were produced, and their catalytic performances were compared. The top-performance bifunctional catalytic material MnO_*x*_/Ti_4_O_7_-300 shows the highest proportion of Mn^3+^. The value of e_g_-filling and the covalent property were utilized to explain this phenomenon theoretically, the results being consistent with the experimental results.

(3) A Li–O_2_ battery based on ORR and OER catalysis was assembled. It was proved that MnO_*x*_/Ti_4_O_7_-300 shows bifunctional catalytic performance and charge/discharge property in the Li–O_2_ battery, which indicates that the MnO_*x*_/Ti_4_O_7_ catalytic material possesses application prospect.

## Conflicts of interest

There are no conflicts to declare.

## Supplementary Material

RA-011-D0RA08575H-s001
