# Supplementary material for: Effect of the valence state of Mn in MnOx/Ti4O7 composites on the catalytic performance for oxygen reduction reaction and oxygen evolution reaction
Source: RSC Adv. 2021 Jan 5;11(3):1524–30. doi: 10.1039/d0ra08575h (PMC8693610; doi:10.1039/d0ra08575h)
Supplement: RA-011-D0RA08575H-s001 [file RA-011-D0RA08575H-s001.pdf]

Electronic Supplementary Material (ESI) for RSC Advances.
This journal is © The Royal Society of Chemistry 2020

Effect on valence state of Mn in MnO_x/Ti_4O_7 composites for catalytic performance on oxygen reduction reaction and oxygen evolution reaction

Fan Bai,[a] Lincheng Xu,[ab] Daode Wang,[a] Li An,*[c] Zhanzhong Hao*[b] and Fan Li*[a]

a. Beijing Key Laboratory for Catalysis and Separation, Department of Environment and Chemical Engineering, Beijing University of Technology, Beijing 100124, China. E-mail: vanadiumli@bjut.edu.cn
b. Department of Chemistry, Baotou Teachers' college, Baotou 014000, China. E-mail: 18438602389@163.com
c. Beijing Key Laboratory for Catalysis and Separation, Faculty of Environment and Life, Beijing University of Technology, Beijing 100124, China. E-mail: 08131@bjut.edu.cn

*Corresponding Authors
E-mail: 08131@bjut.edu.cn;
18438602389@163.com;
vanadiumli@bjut.edu.cn.

Electrochemical measurements

All of the electrochemical measurements were carried with a three-electrode system, including working electrode, reference electrode and rod counter electrode. The working electrode was chosen by different electrochemical measurements. For the cyclic voltammetry (CV), linear sweep voltammetry (LSV) and electrochemical impedance spectroscope (EIS) tests, a glassy carbon working electrode of diameter Φ = 5 mm controlled by the rotating disk electrode (RDE, Pine AFMSRCE or IPS RRDE). As for accelerated durability test (ADT), chronoamperometry (CA) and polarization curves tests, a glassy carbon piece of 10*10 mm was used as working electrode. A Hg/HgO reference electrode (in 0.1 mol/L KOH, 0.165 V vs. RHE) was used as reference electrode and a carbon rod counter electrode (Φ = 2 mm) was used as counter electrode. A customized five-holes flask was used as electrolytic cell, filled with 0.1 mol/L KOH aqueous solution as electrolyte. The electrolyte was saturated in argon firstly in order to activate the system and then saturated in oxygen for further electrochemical measurements. All the electrochemical measurements in this work were without iR compensation.

The CV and LSV tests were measured with saturated in oxygen 0.1 mol/L KOH aqueous solution at the scan rate of 10 mV/s. For LSV tests, the rotate speed of rotating disk electrode was chosen as 225, 400, 625, 900, 1225, 1600, 2025 and 2500 rpm. The curves of 1600 rpm were chosen for LSV images.

The EIS tests were measured with aerating oxygen continuously by aerator pipe at rotate speed of 1600 rpm.

The ADT was measured through several CV and polarization curves tests at the scan rate of 10 mV/s. The polarization curves tests were measured before and after 5000 cycles of CV tests for comparing ORR and OER catalytic stability of different catalytic material. Oxygen was essential during the whole measured process.

The CA tests were measured at potential of 0.68 V vs. RHE according to the

potential of ORR. Oxygen was essential during the measured process.

The electrochemical active surface area (ECSA) for each system was estimated from the electrochemical double-layer capacitance (C_{DL}) of the catalytic surface and specific capacitance (C_S).[S1,S2] The formula was described as eq1:

$$ECSA = C_{DL}/C_S \qquad \text{eq1}$$

As for C_S, it can be regarded as function of electrolyte system and adopted as 0.05 mF/cm^2.[S3,S4] To measure C_{DL}, a potential range in which non-Faradaic processes occur is determined from CV tests. The range is typically a 0.1 V potential window centered at the open-circuit potential (OCP) of the measured system. The formula was described as eq2:

$$i = \nu * C_{DL} \qquad \text{eq2}$$

where i is double-layer current and ν is scan rate. Therefore, the CV tests were measured at range of 0.752~0.872 V vs. RHE (OCP 0.812 V vs. RHE) in figure S6. The scan rates were chosen as 10, 20, 40, 60, 80 and 100 mV/s and i was current at the potential of 0.812 V vs. RHE. A series of plots were described as i-ν curves with a slope equal to C_{DL} as shown in figure S7.

The K-L equation can be calculated by eq3:

$$1/J = 1/J_L + 1/J_K = 1/(B\omega^{1/2}) + 1/J_K \qquad \text{eq3}$$

where J is current density at a certain potential, J_L is dynamic current density, J_K limiting diffusion current density, ω is rotate speed and B is function of electron transfer numbers n. A series of plots were described as $(1/J)$-$(1/\omega^{1/2})$ curves with a slope equal to 1/B and then electron transfer numbers n can be calculated. The K-L equation of different catalytic material were shown in figure S7 at the rotate speed of 225, 400, 625, 900, 1225, 1600, 2025 and 2500 rpm and scan rate of 10 mV/s.

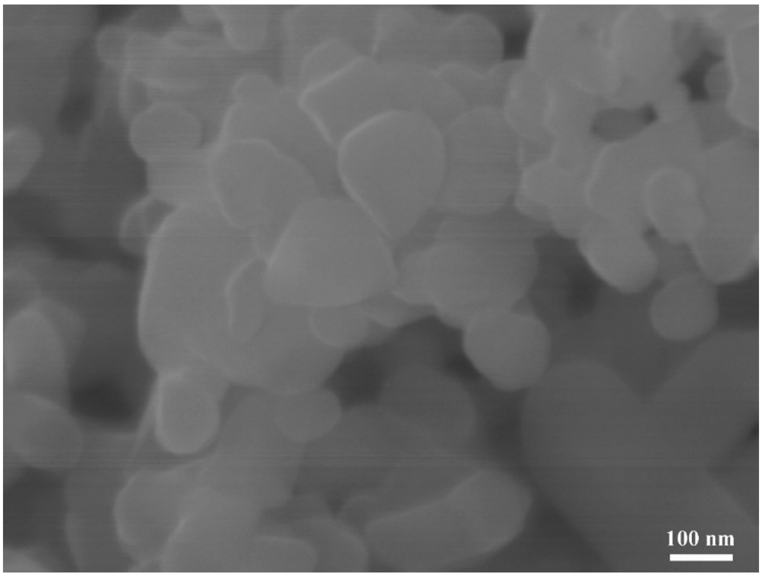

Figure S1. SEM image of Ti_4O_7.

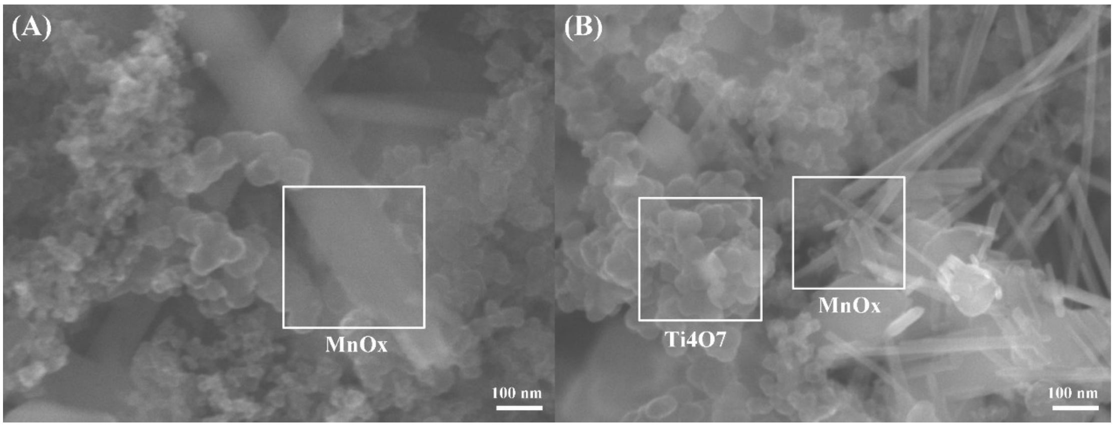

Figure S2. High-magnification SEM images of samples (A) MnO_x and (B) MnO_x/Ti_4O_7.

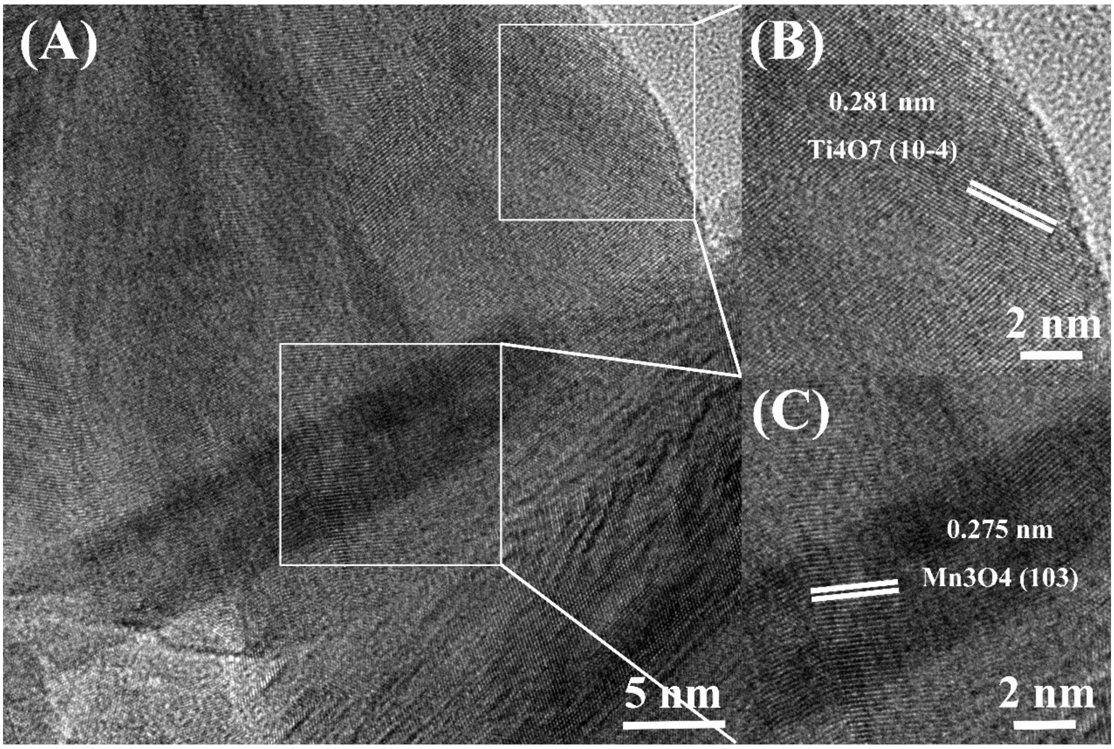

Figure S3. HRTEM images of (A) MnO_x/Ti_4O_7; lattice plane of (B) Ti_4O_7(10-4) and (C) Mn_3O_4(103).

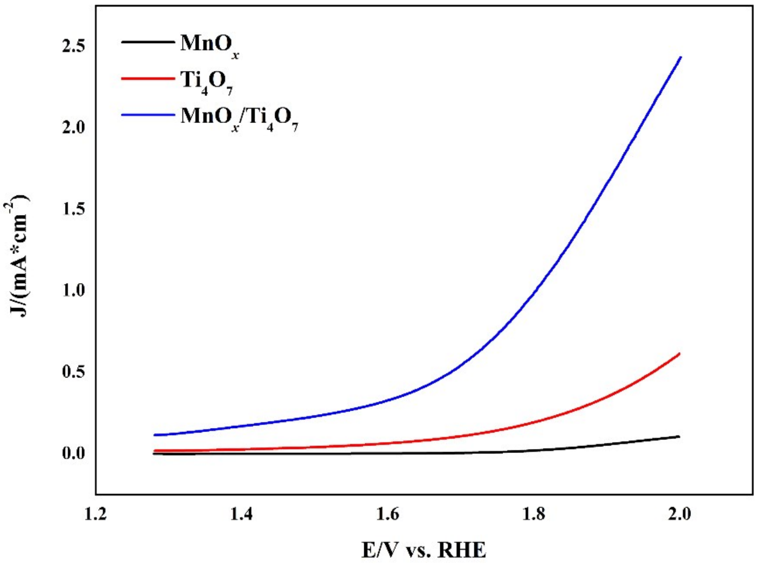

Figure S4. LSV curves of Ti_4O_7, MnO_x and MnO_x/Ti_4O_7 for OER at 1600 rpm, scan rate of 10 mV/s.

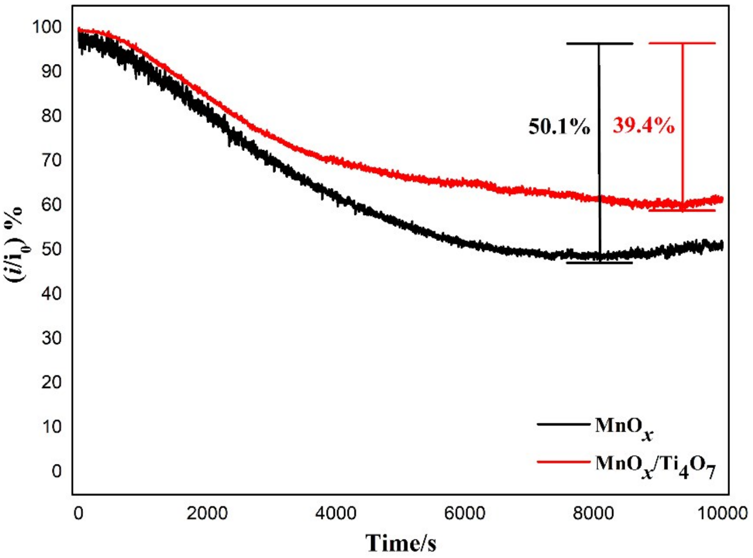

Figure S5. CA curves of MnO_x and MnO_x/Ti_4O_7 at potential of 0.68 V vs. RHE for ORR durability test.

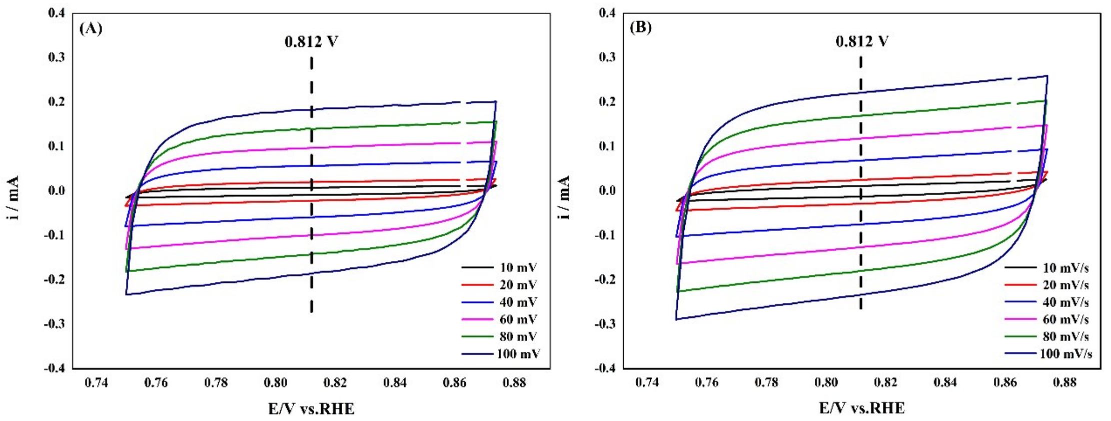

Figure S6. CV curves in a non-Faradaic region of (A) MnO_x and (B) MnO_x/Ti_4O_7 at scan rates of 10, 20, 40, 60, 80 and 100 mV/s for C_{DL} and ECSA measurements.

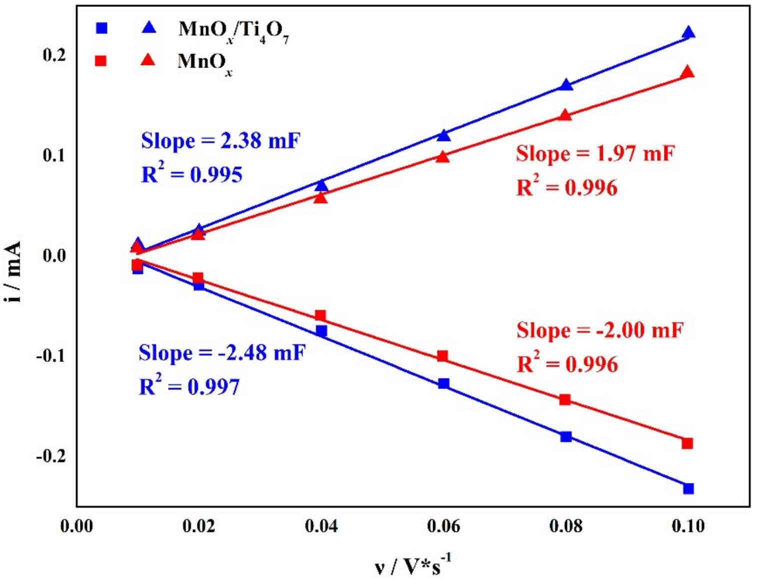

Figure S7. The cathodic and anodic currents MnO_x and MnO_x/Ti_4O_7 measured at 0.812 V vs. RHE as a function of scan rates. C_{DL} value of the system is determined by the average of the slope absolute value of the linear fits to the data.

Table S1. Comparsion of ORR/OER catalytic performance for benchmark noble metal and manganese oxides based catalysts.

Catalysts	Electrolyte	Half-wave potential for ORR (V)	Overpotential for OER at 10 mA/cm^2 (mV)	Ref.
Pt/C	0.1 M KOH	0.82	--	S5
PtGa NWs	0.1 M $HClO_4$	0.86	--	S6
IrO_2	1.0 M KOH	--	255	S7
RuO_2	0.1 M KOH	--	390	S8
NiFe-LDHs	1.0 M KOH	--	190	S9
α-MnO_2-SF	0.1 M KOH	0.78	490	S10
Mn_3O_4/Ti_3C_2	0.1 M KOH	0.85	--	S11
Mn_3O_4-NR	1.0 M NaOH	0.78	--	S12
Mn_3O_4	0.1 M KOH	--	Not reaching	S13
Mn_3O_4	0.1 M KOH	0.63	--	S14
Hollow Mn_2O_3	0.1 M NaOH	0.71	--	S15
MnO_x/Ti_4O_7-300	0.1 M KOH	0.72	670	This work

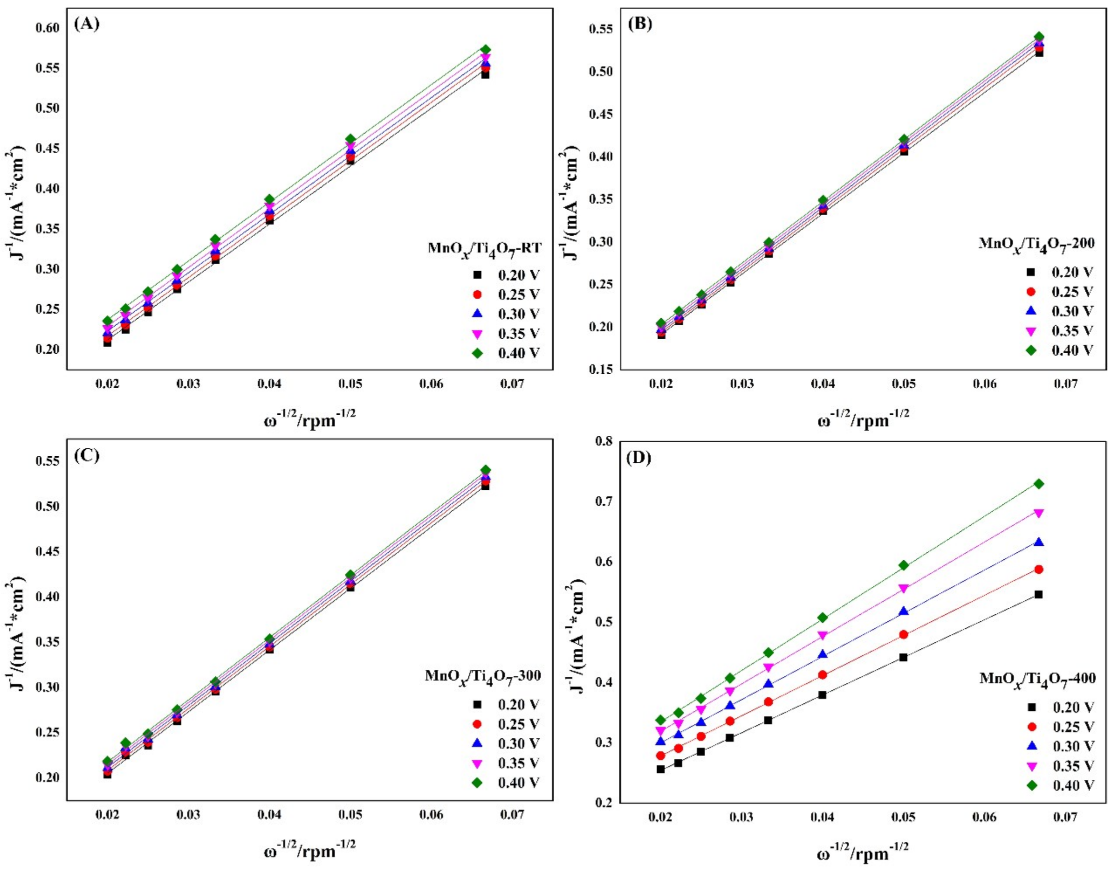

Figure S8. K-L equation of (A) MnO_x/Ti_4O_7-RT, (B) MnO_x/Ti_4O_7-200, (C) MnO_x/Ti_4O_7-300 and (D) MnO_x/Ti_4O_7-400 for measuring ORR electron transfer numbers at 225, 400, 625, 900, 1225, 1600, 2025 and 2500 rpm.

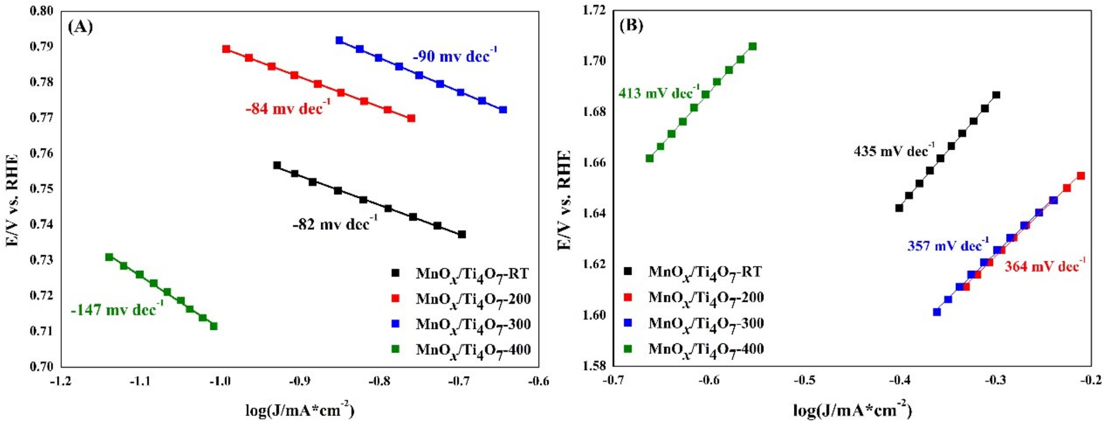

Figure S9. Tafel curves of MnO_x/Ti_4O_7-RT, MnO_x/Ti_4O_7-200, MnO_x/Ti_4O_7-300 and MnO_x/Ti_4O_7-400 for (A) ORR and (B) OER range.

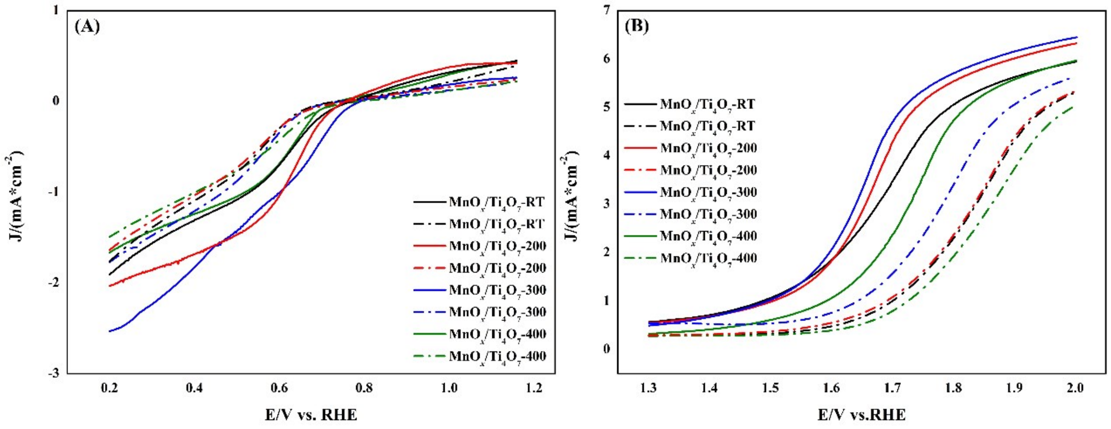

Figure S10. (A) ORR and (B) OER polarization curves of MnO_x/Ti_4O_7-RT, MnO_x/Ti_4O_7-200, MnO_x/Ti_4O_7-300 and MnO_x/Ti_4O_7-400 before (full line) and after (dotted line) 5000 CV cycles.

References

S1 S. Trasatti, O. A. Petrii, *Pure Appl. Chem*. 1991, **63**, 711-734.

S2 C. C. L. McCrory, S. Jung, J. C. Peters and T. F. Jaramillo, *J Am Chem Soc*, 2013, **135**, 16977-16987.

S3 K. Zeng, H. Yu, Z. Sun, J. Yan, X. Zheng, Y. Jiang, W. Hu, J. Tian and R. Yang, *ACS Appl. Mater. Interfaces*, 2020, **12**, 39205-39214.

S4 P. Connor, J. Schuch, B. Kaiser and W. Jaegermann, *Z. Phys. Chem*, 2020; **234**, 979-994.

S5 P. Chen, T. Zhou, L. Xing, K. Xu, Y. Tong, H. Xie, L. Zhang, W. Yan, W. Chu, C. Wu and Y. Xie, *Angew Chem Int Edit*, 2017, **129**, 625-629.

S6 L. Gao, X. Li, Z. Yao, H. Bai, Y. Lu, C. Ma, S. Lu, Z. Peng, J. Yang, A. Pan and H. Huang, *J. Am. Chem. Soc*, 2019, **141**, 18083-18090.

S7 W. Zhong, Z. Lin, S. Feng, D. Wang, S. Shen, Q. Zhang, L. Gu, Z. Wang and B. Fang, *Nanoscale*, 2019, **11**, 4407-4413.

S8 M. Gao, W. Sheng, Z. Zhuang, Q. Fang, S. Gu, J. Jiang and Y. Yan, *J Am Chem Soc*, 2014, **136**, 7077-7084.

S9 S. Jiao, Z. Yao, M. Li, C. Mu, H. Liang, Y. Zeng and H. Huang, *Nanoscale*, 2019, **11**, 18894-18899.

S10 Y. Meng, W. Song, H. Huang, Z. Ren, S. Chen and S. L. Suib, *J Am Chem Soc*, 2014, **136**, 11452-11464.

S11 Q. Xue, Z. Pei, Y. Huang, M. Zhu, Z. Tang, H. Li, Y. Huang, N Li, H. Zhang and C. Zhi, *J Mater Chem A*, 2017, **5**, 20818-20823.

S12 J. Liu, L. Jiang, T. Zhang, J. Jin, L. Yuan and G. Sun, *Electrochim Acta*, 2016, **205**, 38-44.

S13 S. Hirai, S. Yagi, A. Seno, M. Fujioka, T. Ohno and T. Matsuda, *Rsc Adv*, 2016, **6**, 2019-2023.

S14 W. Wang, J. Geng, L. Kuai, M. Li and B. Geng, *Chem-Eur J*, 2016, **22**, 9909-9913.

S15 N. Parveen, Z. Khan, S. A. Ansari, S. Park, S. T. Senthilkumar, Y. Kim, H. Ko and M. H. Cho, *Chem Eng J*, 2019, **360**, 415-422.
